# Big Bubble Deep Anterior Lamellar Keratoplasty for Management of Deep Fungal Keratitis

**DOI:** 10.1155/2014/209759

**Published:** 2014-07-01

**Authors:** Hua Gao, Peng Song, Jose J. Echegaray, Yanni Jia, Suxia Li, Man Du, Victor L. Perez, Weiyun Shi

**Affiliations:** ^1^Shandong Eye Hospital, Shandong Eye Institute, Shandong Academy of Medical Sciences, 372 Jingsi Road, Jinan 250021, China; ^2^Bascom Palmer Eye Institute, University of Miami, 1638 NW 10th Avenue, Miami, FL 33136, USA; ^3^Shandong Eye Institute, 5 Yanerdao Road, Qingdao 266071, China

## Abstract

*Objective*. To evaluate the therapeutic effect of big bubble deep anterior lamellar keratoplasty (DALK) in patients with deep fungal keratitis. *Methods*.Consecutive patients who had DALK for deep fungal keratitis at Shandong Eye Hospital between July 2011 and December 2012 were included. In all patients, the infiltration depth was more than 4/5ths of the corneal thickness. DALK surgery was performed with bare Descemet membrane (DM) using the big bubble technique. Corrected distance visual acuity (CDVA), graft status, and intraoperative and postoperative complications were monitored. *Results*. Big bubble DALK was performed in 23 patients (23 eyes). Intraoperative perforation of the DM occurred in two eyes (8.7%) during stromal dissection. The patients received lamellar keratoplasty with an air bubble injected into the anterior chamber. Double anterior chamber formed in 3 eyes (13.0%). Mean CDVA of the patients without cataract, amblyopia, and fungal recurrence was improved from preoperative HM/20 cm−1.0 (LogMAR) to 0.23 ± 0.13 (LogMAR) at the last followup (*P* < 0.01). Fungal recurrence was found in two patients (8.7%). Corneal stromal graft rejection was noted in one patient (4.3%). *Conclusions*. DALK using the big bubble technique seems to be effective and safe in the treatment of deep fungal keratitis unresponsive to medication.

## 1. Introduction

Fungal keratitis (FK) is a major blinding eye disease in Asia [1] and is becoming the first indication for corneal transplantation in China [2, 3]. Due to difference in pathogenic species, glucocorticoid abuse, diagnostic delay, lack of antifungal agents, and low drug sensitivities, many patients in China showed more severe symptoms compared to patients in Europe and the United States [2–5].

When patients are unresponsive to antifungal medication, surgical treatment should be considered to preserve the eye globe and improve visual acuity [1, 6, 7]. Penetrating keratoplasty (PK) and lamellar keratoplasty (LK) have been employed for management of FK [4, 8], but complications such as immune graft rejection after PK [6, 9] and interface haze after LK may impede visual acuity recovery [10]. For cases with deep infection, it seems to be more prone to recurrence after LK [11], while PK surgery becomes a relatively popular choice for full-thickness resection of the infected tissue.

Deep anterior lamellar keratoplasty (DALK) is currently considered to be the first option by many corneal surgeons for patients with corneal diseases not involving the endothelial layer due to its satisfactory clinical results compared with PK [12–15]. However, few surgeons apply it for management of infectious corneal ulcers. Our present study aimed to evaluate the outcomes of big bubble DALK in patients with deep FK unresponsive to antifungal medication.

## 2. Materials and Methods

### 2.1. Patients

This study was approved by the ethics committee of Shandong Eye Institute. Medical records of patients who were treated by big bubble DALK for deep FK at Shandong Eye Hospital between July 2011 and December 2012 were reviewed. All patients had antifungal medication for at least 2 weeks. If the infection did not heal, DALK surgery was considered in patients with infection or infiltrates penetrating greater than 4/5ths of the corneal thickness in the deepest area as observed by slit-lamp microscopy and laser scanning confocal microscopy (Heidelberg Engineering GmbH), respectively. Those with combined perforation were excluded from this study. Informed consent was obtained from the patients involved.

### 2.2. Surgical Technique

**Figure 1:**
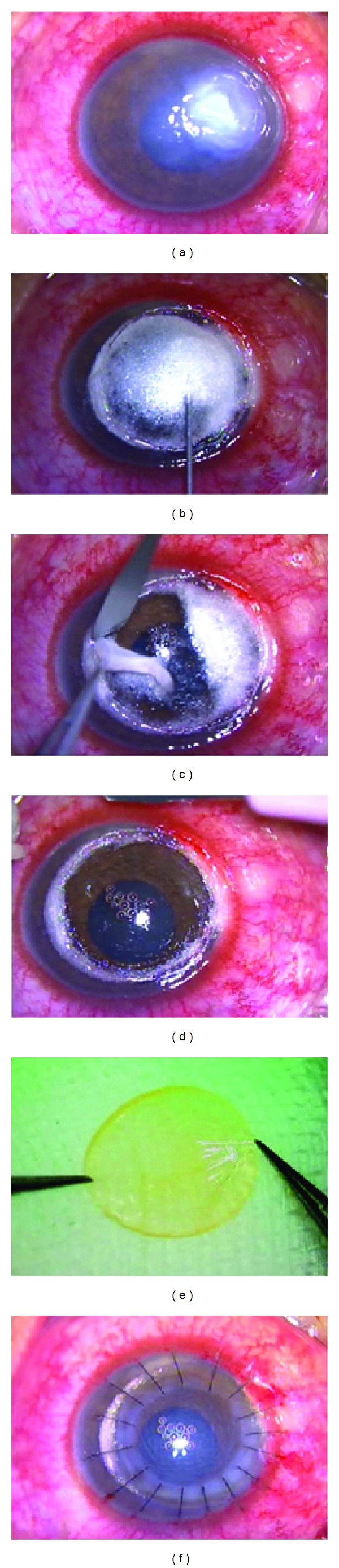
Surgical procedure of deep anterior lamellar keratoplasty for deep fungal keratitis. (a) Deep fungal ulceration with hypopyon before surgery. (b) A big bubble formed after 1.5 mL sterilized air is injected into the posterior stroma. (c) Debulking of the posterior stroma is performed with a 45° micro knife. (d) Descemet membrane is bared after the diseased stoma was cut off. (e) The donor Descemet membrane and endothelium are stripped off using 0.12 mm untoothed forceps. (f) The donor cornea is sutured to the recipient with 16 interrupted 10/0 nylon sutures.

All surgeries were performed by a single surgeon (H. G.) under peribulbar anesthesia (2% lignocaine hydrochloride and 1% ropivacaine) using the big bubble technique (Figure 1). Briefly, a Hessburg-Barron vacuum trephine (Katena, Denville, New Jersey, USA) was used to make a partial thickness trephination (about 300 *μ*m) on the host cornea, and then the anterior diseased stroma was cut off. A 30-gauge disposable needle attached to a 2 mL syringe and bent at 15–30° was advanced and beveled down into the paracentral corneal stroma. About 1–1.5 mL of sterilized air was injected into the posterior stroma until a big bubble was formed extending to the border of trephination. If the big bubble did not form after the first attempt, the injection was repeated. After the big bubble formation, debulking of the white posterior stroma was performed with a 45° micro knife (Alcon Laboratories, Fort Worth, Texas, USA), leaving a very thin layer of corneal stromal tissue over the air bubble. Thereafter, a peripheral paracentesis was performed to reduce intraocular pressure. A small opening was created in the stromal tissue overlying the air bubble using a 30-gauge disposable needle. After an iris repositor was employed to retract the residual stroma, a 45° blade was used to divide the rest of the corneal stroma into 2 parts. The stroma was then held using toothed forceps, and the residual stroma was cut off using a 45° blade along the line of the trephination groove to bare the Descemet membrane (DM). The recipient bed was ready.

Full-thickness donor corneal tissues stored in D-X medium at 4°C or in glycerin at −20°C were used for transplantation. The donor cornea was punched from the endothelial side using the Barron punch (Katena, Denville, New Jersey, USA) and was oversized by 0.25 mm. The donor DM and endothelium were gently stripped off using 0.12 mm untoothed forceps, which could prevent wrinkling of the donor DM at the interface. After the donor cornea was sutured to the recipient with 16 interrupted 10/0 nylon sutures (Mani, Tochigi, Japan), the tightness of the sutures was adjusted by a Placido disc to reduce postoperative astigmatism when the corneal echogenic ring became relatively round. Finally, 0.2% fluconazole (0.5 mL) was injected subconjunctivally, and 0.3% ofloxacin topical eye ointments (Santen, Osaka, Japan) were used.

### 2.3. Histopathology

Partial corneal buttons (anterior and posterior stroma) obtained during DALK were fixed in 4% formalin and embedded in paraffin. Serial slices (4 *μ*m) were stained with periodic acid-Schiff (PAS). The presence of hyphae and spores was observed by light microscopy.

### 2.4. Perioperative Treatment

Preoperative management included topical 5% natamycin eye drops (q 1 hour; Alcon, Fort Worth, Texas, USA) and 0.2% fluconazole eye drops (q 30 minutes; Shenyang Sinqi Pharmaceutical, Shenyang, China). Both antifungal agents were reduced to 4 times daily after surgery. Systemic fluconazole 0.2 (Cisen Pharmaceutical, Jining, China) was given intravenously every day in patients with hypopyon, and the duration was not longer than 2 weeks.

Postoperatively, 1% cyclosporin A eye drops were given 4 times per day. If no recurrence was detected at 3 weeks after DALK surgery, 0.02% fluorometholone eye drops (Santen Pharmaceutical, Osaka, Japan) were used 3-4 times per day for about 6 months and tapered thereafter.

### 2.5. Perioperative Evaluation

Preoperatively, complete ocular examinations were performed, including uncorrected distance visual acuity (UDVA), corrected distance visual acuity (CDVA), slit-lamp examination, anterior segment optical coherence tomography (AS-OCT), corneal smear, bacterial and fungal cultures, and laser scanning confocal microscopy examination.

Postoperative follow-up examinations were scheduled every week for the first month and once a month thereafter. The main outcome measures were the success of the big bubble technique, intra- and postoperative complications or secondary interventions, CDVA, rejection episode, endothelial cell density, and recipient thickness. The endothelial cell density was measured by specular microscopy, and the recipient thickness was measured by AS-OCT.

### 2.6. Statistical Analysis

SPSS 17.0 was used for statistical analysis. The two-sample *t*-test was used to compare the parameters perioperatively. *P* < 0.05 was considered statistically significant.

## 3. Results

### 3.1. Patient Information

A total of 23 patients (23 eyes) underwent big bubble DALK during the study period (12 males and 11 females). The mean age was 46.9 ± 11.6 years (range: 24 to 72 years). The mean follow-up time was 12.5 ± 2.5 months (range: 9 to 18 months). All cases had deep FK, including 8 cases (34.8%) with hypopyon of 2.3 ± 0.9 mm (range: 0.5 to 3 mm). The mean size of ulcer and stromal infiltrate was 7.0 ± 0.5 mm × 6.3 ± 0.9 mm. The mean diameter of the trephine was 7.8 ± 0.3 mm in the recipient and 8.1 ± 0.3 mm in the donor (Table 1).

### 3.2. Smear, Confocal Microscopy, and Culture Information

The KOH smear was positive in 21 (91.3%) patients with corneal scrapings, and the laser scanning confocal microscopy was positive in 22 (95.7%) patients before surgery. Hyphal infiltration was not found in the DM by confocal microscopy in any patient. Seventeen (73.9%) patients had positive fungal cultures, including 12 patients with* Fusarium*, 2 with* Aspergillus*, 2 with* Agonmycetaceae*, and 1 with* Alternaria *Nees.

### 3.3. Histopathology

**Figure 2:**
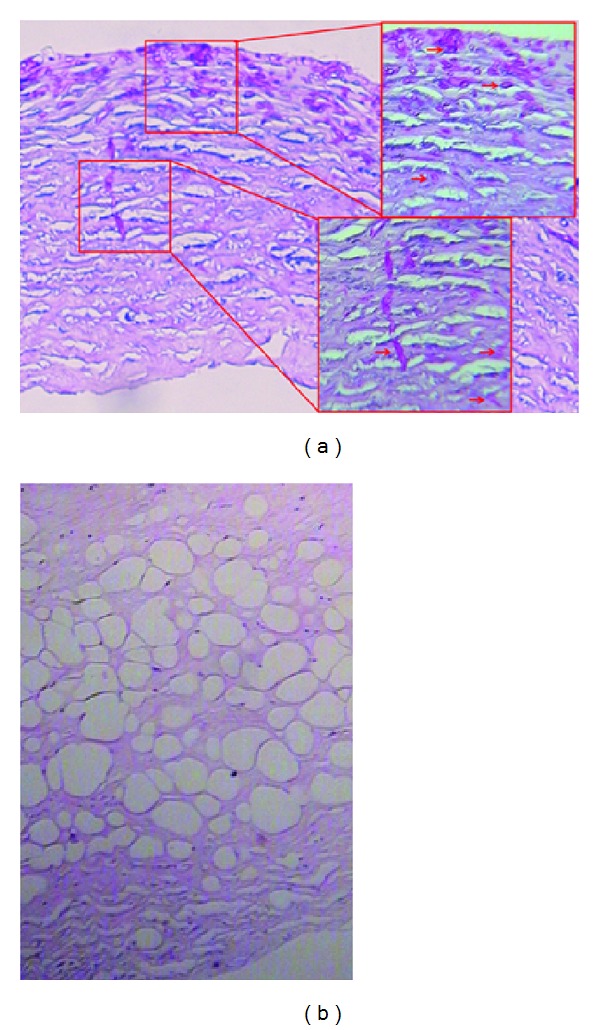
The density of the hyphae and spores (arrows) is much higher in the anterior stroma than that in the posterior stroma (a), ×100. The posterior stroma is loosened due to the air injection, and the hyphae and spores are not seen in the stroma near the Descemet membrane (b), ×100.

The hyphae were observed in 21 cases (91.3%) by histopathology examination. Hyphae and spores invaded into the corneal stroma, and about 1/2 to 2/3 of the stroma was involved in most cases. The density of the hyphae and spores was much higher in the anterior stroma than that in the posterior stroma. The posterior stroma was very loosening due to the air injection. No hyphae or spores were seen in the stroma near DM (Figure 2).

### 3.4. Perioperative Complications

Intraoperative microperforation of the DM occurred in 2 eyes (8.7%) during stromal dissection. These two patients still received LK with an air bubble injected into the anterior chamber.

A double anterior chamber (interface fluid) occurred on postoperative day 1 in 3 patients (13.0%). Among them, one patient had intraoperative DM microperforation and was managed with intracameral injection of air bubble accompanied with pupil dilation. The interface fluid resolved spontaneously within 5 days. In the other two patients, the interface fluid was drained using 0.12 mm blunt forceps to separate the incision or resolved spontaneously within 3 days.

The corneal epithelium healed in all cases within 1 week after surgery. Two patients (8.7%) suffered fungal recurrence within postoperative 3 days. One (case number 14) was unresponsive to antifungal treatment and received secondary PK to control the recurrence. This patient had presented with 2 mm of hypopyon before DALK procedure with cultures identifying* Fusarium oxysporum* as the causative organism. The other with previous cultures identifying* Fusarium moniliforme* as the causative organism was cured with subconjunctival injection of fluconazole (2 mg/mL) twice a day for 10 days.

Corneal stromal graft rejection occurred in 1 case (4.3%) during the first 3 months after surgery. This patient presented with a decrease in visual acuity. Slit-lamp examination showed conjunctival congestion, subepithelial infiltrates, and mild stromal edema. Topical 1% prednisolone acetate was given hourly for 3 days and tapered off over the next 2 weeks. No secondary glaucoma was observed.

### 3.5. Visual Acuity and Recovery

The mean preoperative CDVA was HM/20 cm to 1.0 (LogMAR). The patients (16 cases) without cataract, amblyopia, and fungal recurrence had a mean LogMAR CDVA improvement of 0.34 ± 0.17 (range: 0.15 to 0.70; *P* < 0.01) at 6 months postoperatively. Further improvement was seen at the last follow-up visit when the mean LogMAR CDVA was 0.23 ± 0.13 (range: 0.10 to 0.52; *P* < 0.01). At the last followup, the CDVA was ≥20/40 in 81.3% of the 16 patients and ≥20/66 in all of them. Moreover, the CDVA in 6 patients with cataract and/or amblyopia was improved to 20/200-20/63. The patient with secondary PK after fungal recurrence achieved a CDVA of 20/20. Mean spherical equivalent was −0.84 ± 3.2 D, and mean astigmatism was −2.64 ± 2.40 D (range: −0.5 to −4.25) at 6 months in all patients.

### 3.6. Graft Evaluation

**Figure 3:**
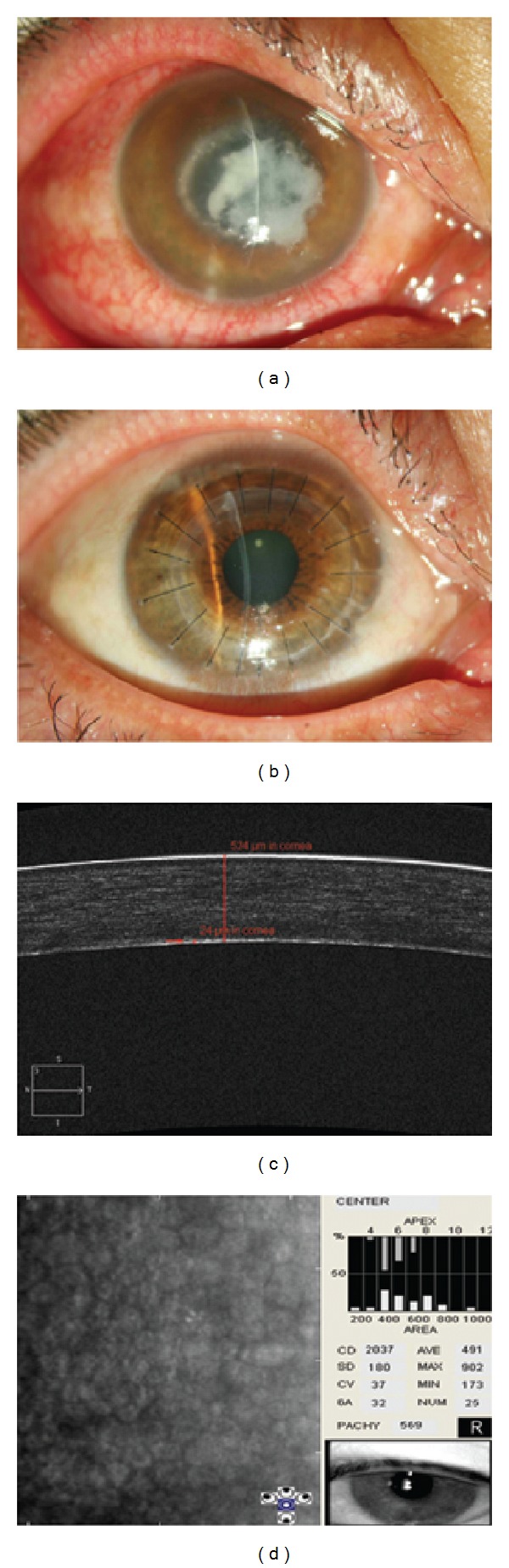
Slit-lamp photographs of fungal keratitis before deep anterior lamellar keratoplasty (CDVA = 0.02; (a)) and after surgery (CDVA = 0.8; (b)). AS-OCT shows that the recipient bed thickness is 24 *μ*m, the graft and recipient match well, and the graft-host interface (red arrow) can hardly be seen (c). The endothelial cell density is 2037 cells/mm^2^ by specular microscopic examination (d).

The entire graft was clear in all patients at the last follow-up visit. The mean recipient bed thickness was 22.5 ± 3.64 *μ*m (range: 16 to 30) as measured by AS-OCT. The grafts and recipients matched well, and the interfaces were hardly noticeable by slit-lamp microscopy and AS-OCT examination. The mean endothelial cell density in the central area of 22 patients who accepted DALK successfully was 2120 ± 461 (range: 1359 to 2994) cells/mm^2^ (Figure 3). The endothelial cell density of the patient who received secondary PK decreased from 1727 cells/mm^2^ (2 months postoperatively) to 1145 cells/mm^2^ (18 months postoperatively).

## 4. Discussion

According to the World Health Organization, infectious corneal diseases are a major cause of blindness worldwide, second only to cataract in overall prevalence. Among severe infective corneal ulcers, FK is most common in many developing countries like China, India, Ghana, and Nepal [2, 16, 17].

Due to limited options of commercially available antifungal drugs and low drug sensitivity in some patients, surgical treatment is required to preserve the patient's eyeball and restore useful vision in severe cases. This is especially true when antifungal therapy fails to control the infection [4, 18].

In the early stage, most ophthalmologists thought that fungal hyphae in the stroma grew perpendicular to the corneal stromal collagen, and penetration of the hyphae to the corneal endothelium may result in perforation. In such instances, LK is not adequate to completely remove the infected tissue, and PK may be the only option to control the fungal infection. Sedghipour et al. [19] and Said et al. [7] reported satisfactory results after PK for the treatment of FK, but the postoperative immune rejection was high (27.2%–29.6%), and the long-term outcomes were not favorable [19, 20].

With further understanding of FK and advances in microsurgical techniques, LK has been found to be effective in the treatment of FK before the hyphae penetrate the full-thickness cornea, with a decreased risk of immune rejection and graft dehiscence [8]. But for deep infection, LK may increase the risk of recurrence after surgery if the excision of the ulcer is not complete [21]. Moreover, fiber formation in the irregular interface can affect the postoperative visual recovery [10]. Therefore, an approach of dissecting the whole corneal stroma may be helpful in these kinds of patients.

Big bubble DALK was first introduced by Anwar and Teichmann in 2002 [22]. Keratoconus was one of the major indications for DALK initially [23, 24]. Over the past few years, DALK procedures have been performed to treat corneal stromal diseases like corneal dystrophies, corneal ectasia, corneal scar [13–15, 25], and even infectious keratitis [12]. Anshu and his colleagues compared the therapeutic effects of DALK and PK for advanced bacterial, fungal, and* Acanthamoeba* keratitis, finding that DALK can result in better graft survival and visual outcomes [12]. Considering the advantages of DALK surgery, we used it in the management of deep FK unresponsive to antifungal treatment in this study. Although the infection or infiltration in our patients was very deep and 8 (34.8%) of them even had hypopyon, we found that the density of the hyphae and spores was much lower in the posterior stroma than that in the anterior stroma, and no hyphae or spores were detected in the posterior stroma near the DM by the histology examination after surgery. Therefore, DALK procedure had the potential to clear the hyphae and spores, even in patients with deep infectious keratitis. Moreover, theoretically this procedure might decrease fungal recurrence more significantly than traditional LK. According to preoperative slit-lamp and confocal microscopic examinations, as well as clear intraoperative observation of the recipient, surgeons can better determine if the hyphae spread in the full thickness of the cornea and if the patient should receive DALK.

In DALK, the pathologic corneal stroma is replaced, while the healthy endothelium of the host is preserved. This helps to retain all the advantages of anterior lamellar keratoplasty over full-thickness keratoplasty, providing a clearer interface. In this study, 81.3% of the patients had a final CDVA of ≥20/40 after DALK, which is similar to the results of PK in the treatment of infectious keratitis [12, 26] and PK or DALK in the treatment of noninfectious corneal disease, such as keratoconus and corneal dystrophies [12, 15, 24]. In addition, DALK procedure avoids most of the complications associated with an open-sky surgery and grossly avoids postoperative endothelial rejection.

Intraoperative perforation of DM is one of the most common complications during DALK surgery, with a rate of 9% to 23% [27, 28]. In cases with FK, the stroma usually has edema, which allows more adequate intraoperative stromal dissection compared to patients with stromal scarring [29]. Therefore, perforation in such patients is not common. Management of DM perforation depends on the size and location of the perforation. Macroperforations may require conversion to a full-thickness keratoplasty, but microperforations allow completion of DALK or LK in the majority of cases [27]. Although intraoperative perforation of the DM occurred in 2 (8.7%) eyes in our series, LK was still performed successfully in these two patients with an air bubble injected into the anterior chamber.

Double anterior chamber is another common complication after the DALK surgery [27, 29]. This can occur with development of DM perforation or with transient endothelial dysfunction due to tight sutures in the presence of graft edema. In this study, 3 cases presented with double anterior chamber postoperatively. One eye was managed by tamponade with intracameral gas, and 2 eyes resolved spontaneously. Recurrence may be one of the postoperative complications after keratoplasty for FK, with a rate of 7.6%–20.0% [12, 20, 30]. In our study, recurrence was found in only two patients (8.7%), suggesting that DALK does not increase the risk of disease recurrence. Immune rejection was not a major complication in our study. This may be associated with the donor's lowered antigenicity when the graft lacked an endothelium. The low immune rejection incidence after lamellar keratoplasty or therapeutic DALK in the treatment of infectious keratitis was previously reported by Xie et al. [8] and Anshu et al. [12].

In conclusion, DALK using the big bubble technique appears to be effective in the treatment of deep FK that is unresponsive to antifungal treatment. This approach can not only decrease the risk of rejection episodes, but also provide a clear interface between the recipient and the graft, achieving satisfactory visual acuities.

## Figures and Tables

**Table 1:** Clinical profiles of fungal keratitis after deep anterior lamellar keratoplasty.

No.	Age/gender/infected eye	Preoperative CDVA	Infiltrate area (mm)	Hypopyon (mm)	Confocal	Smear	Culture information	Recipient/graft diameter (mm)	Complications	Accompanied diseases	ECD (cells/mm^2^)	CRT (*μ*m)	CDVA at last follow up
1	48/M/OD	CF/10 cm	7.0 ∗ 6.5	3 mm	Positive	Positive	Negative	7.75/8.0	Interface fluid	No	1359	23	20/40
2	51/F/OS	20/200	7.5 ∗ 6.5	No	Positive	Positive	Negative	7.75/8.25	No	No	2242	28	20/40
3	59/F/OS	HM/20 cm	7.5 ∗ 7.0	3 mm	Positive	Negative	Negative	8.0/8.25	No	Cataract	NA	24	20/63
4	44/M/OD	CF/30 cm	7.0 ∗ 7.0	No	Positive	Positive	Aspergillus	7.75/8.0	No	No	2273	24	20/32
5	26/F/OS	20/1000	8.0 ∗ 8.0	No	Positive	Positive	Agonmycetaceae	8.75/9.0	No	No	2037	24	20/25
6	37/F/OS	CF/10 cm	7.0 ∗ 6.5	No	Positive	Positive	Fusarium	7.75/8.0	No	No	2101	19	20/63
7	37/F/OD	20/200	7.5 ∗ 7.0	No	Positive	Positive	Negative	7.75/8.0	Interface fluid	No	2433	30	20/40
8	43/M/OS	CF/10 cm	7.5 ∗ 7.5	0.5 mm	Positive	Positive	Fusarium	8.0/8.25	Graft rejection	No	2262	21	20/50
9	46/M/OD	20/250	7.0 ∗ 7.0	No	Positive	Positive	Negative	7.5/7.75	Intraoperative microperforation	No	NA	18	20/25
10	65/M/OS	20/400	7.0 ∗ 7.0	No	Positive	Positive	Alternaria Nees	7.75/8.0	No	Cataract	1958	28	20/63
11	55/M/OS	20/500	7.0 ∗ 6.0	No	Positive	Positive	Fusarium	7.5/7.75	Recurrence	No	1381	20	20/32
12	49/F/OS	HM/20 cm	7.0 ∗ 6.0	No	Positive	Positive	Agonmycetaceae	7.75/8.0	Interface fluid	No	2817	21	20/25
13	60/M/OD	20/500	6.5 ∗ 6.0	2 mm	Positive	Negative	Negative	7.5/7.75	No	Cataract	NA	25	20/63
14	33/M/OD	CF/10 cm	7.0 ∗ 7.0	2 mm	Positive	Positive	Fusarium	7.75/8.25	Recurrence	No	1145	20	20/20
15	37/M/OD	HM/20 cm	7.5 ∗ 5.0	No	Positive	Positive	Fusarium	8.0/8.25	No	No	2755	24	20/25
16	56/M/OS	HM/30 cm	7.0 ∗ 6.0	No	Positive	Positive	Fusarium	7.75/8.0	No	Amblyopia	1859	22	20/200
17	42/M/OS	CF/30 cm	5.5 ∗ 5.0	No	Positive	Positive	Fusarium	7.75/8.0	No	No	NA	17	20/50
18	49/F/OD	20/200	7.0 ∗ 6.0	No	Positive	Positive	Fusarium	7.75/8.0	No	No	1949	19	20/32
19	49/F/OS	HM/20 cm	7.0 ∗ 6.5	3 mm	Positive	Positive	Fusarium	7.5/7.75	No	Cataract	NA	24	20/100
20	72/F/OD	HM/20 cm	6.5 ∗ 6.5	* 无 *	Positive	Positive	Fusarium	7.75/8.0	Intraoperative microperforation	Cataract	1560	22	20/100
21	49/M/OD	HM/40 cm	7.0 ∗ 5.0	2 mm	Negative	Positive	Aspergillus	7.5/7.75	No	No	2994	21	20/32
22	24/F/OS	HM/20 cm	7.0 ∗ 4.0	No	Positive	Positive	Fusarium	7.75/8.25	No	No	1991	16	20/25
23	47/F/OS	20/333	6.5 ∗ 6.5	3 mm	Positive	Positive	Fusarium	7.75/8.0	No	No	2064	27	20/25

M: male; F: female; CDVA: corrected distance visual acuity; CF: counting fingers; HM: hand motion; NA: not available; CRT: central recipient thickness.
